# Critical Role of Non-Halogenated Solvent Additives in Eco-Friendly and Efficient All-Polymer Solar Cells

**DOI:** 10.3390/polym15061354

**Published:** 2023-03-08

**Authors:** Saeah Kim, Huijeong Choi, Myeongjae Lee, Hyeseung Jung, Yukyung Shin, Seul Lee, Kyungkon Kim, Myung Hwa Kim, Kyungwon Kwak, BongSoo Kim

**Affiliations:** 1 Department of Chemistry & Nano Science, Ewha University, Seoul 03760, Republic of Korea; 2 Department of Chemistry, Ulsan National Institute of Science and Technology (UNIST), Ulsan 44919, Republic of Korea; 3 Department of Chemistry, Korea University, Seoul 02841, Republic of Korea; 4 Graduate School of Semiconductor Materials and Device Engineering, Ulsan National Institute of Science and Technology (UNIST), Ulsan 44919, Republic of Korea; 5 Graduate School of Carbon Neutrality, Ulsan National Institute of Science and Technology (UNIST), Ulsan 44919, Republic of Korea

**Keywords:** all-polymer solar cells, non-halogenated solvents, additives, charge lifetime, morphology

## Abstract

Organic solar cells (OSCs) demonstrating high power conversion efficiencies have been mostly fabricated using halogenated solvents, which are highly toxic and harmful to humans and the environment. Recently, non-halogenated solvents have emerged as a potential alternative. However, there has been limited success in attaining an optimal morphology when non-halogenated solvents (typically *o*-xylene (XY)) were used. To address this issue, we studied the dependence of the photovoltaic properties of all-polymer solar cells (APSCs) on various high-boiling-point non-halogenated additives. We synthesized PTB7-Th and PNDI2HD-T polymers that are soluble in XY and fabricated PTB7-Th:PNDI2HD-T-based APSCs using XY with five additives: 1,2,4-trimethylbenzene (TMB), indane (IN), tetralin (TN), diphenyl ether (DPE), and dibenzyl ether (DBE). The photovoltaic performance was determined in the following order: XY + IN < XY + TMB < XY + DBE ≤ XY only < XY + DPE < XY + TN. Interestingly, all APSCs processed with an XY solvent system had better photovoltaic properties than APSCs processed with chloroform solution containing 1,8-diiodooctane (CF + DIO). The key reasons for these differences were unraveled using transient photovoltage and two-dimensional grazing incidence X-ray diffraction experiments. The charge lifetimes of APSCs based on XY + TN and XY + DPE were the longest, and their long lifetime was strongly associated with the polymer blend film morphology; the polymer domain sizes were in the nanoscale range, and the blend film surfaces were smoother, as the PTB7-Th polymer domains assumed an untangled, evenly distributed, and internetworked morphology. Our results demonstrate that the use of an additive with an optimal boiling point facilitates the development of polymer blends with a favorable morphology and can contribute to the widespread use of eco-friendly APSCs.

## 1. Introduction

Organic solar cells (OSCs), such as flexible or semitransparent solar cells, have been widely investigated as a future clean energy source [1,2,3]. An OSC consists of a bulk heterojunction (BHJ)-type photoactive layer, i.e., a blend of *p*-type and *n*-type organic materials. When light enters the photovoltaic layer, photoenergy is converted into electricity by following four steps: (1) exciton generation, (2) exciton diffusion, (3) exciton dissociation and charge generation, and (4) charge transport and charge collection [4,5]. Although OSCs based on *n*-type small molecules have recently achieved high power conversion efficiencies (PCEs) above 18% [6,7,8,9,10,11,12,13,14,15,16], all-polymer solar cells (APSCs) have also attracted considerable attention because of the advantages of *n*-type polymers. The light absorption region and frontier energy levels of *n*-type polymers can be easily tuned to achieve complementary light absorption and a high open-circuit voltage (*V*_oc_) when they are blended with an appropriate *p*-type polymer [17,18]. In comparison with *n*-type small molecules, well-designed *n*-type polymers can have higher mechanical stability, photostability, and thermal stability [19,20,21]. For these reasons, various types of *n*-type polymers have been developed, and APSCs with PCEs over 16% have been reported recently [22,23,24,25].

Despite these advantages, APSCs exhibiting high PCEs are commonly fabricated using halogenated solvents such as chloroform (CF) and chlorobenzene (CB), which are highly harmful to humans and the environment. These toxic halogenated solvents should be replaced with eco-friendly, non-halogenated solvents. There are limited reports on the fabrication of APSCs using non-halogenated solvents because they have poor solubility on conjugated polymers; thus, polymer blend films processed with non-halogenated solvents tend to have large aggregations [26,27,28]. It is crucial to identify effective non-chlorinated solvent systems that can provide sufficient solubility and generate polymer blends with a favorable morphology.

An appropriate non-halogenated solvent system can be identified using *o*-xylene (XY) with non-halogenated additives. XY has a boiling point (bp) of 144 °C and has considerable solvating power, allowing the processability of conjugated polymers. For fullerene acceptor-based OSCs, the successful use of additives was first demonstrated by Heeger’s group with 1,8-octanedithiol [29,30]. In the case of non-halogenated solvent systems, Hou’s group reported that solar cells based on XY + *N*-methyl pyrrolidone (NMP) and anisole + diphenyl ether (DPE) exhibited photovoltaic properties similar to those of solar cells based on a halogenated solvent system of *o*-dichlorobenzene (DCB) + 1,8-diiodooctane (DIO) [31,32]. Yan’s group reported that the 1,2,4-trimethylbenzene (TMB) + 1-phenylnaphthalene (PN) system was more efficient than the CB + DIO system [33]. For *n*-type polymer-based solar cells, the successful use of non-halogenated additives is still rare [27,34,35,36].

Here, we report the photovoltaic properties of PTB7-Th:PNDI2HD-T-based APSCs fabricated using non-halogenated *o*-xylene solvent and various high-boiling-point additives: TMB (bp = 169 °C), indane (IN, 176.5 °C), tetralin (TN, 207 °C), DPE (258 °C), and dibenzyl ether (DBE, 298 °C). These five additives were chosen because they are non-halogenated solvents with a range of boiling points; thus, we can investigate the relationship between the photovoltaic properties and boiling points of the additives. Notably, compared with APSCs processed with a halogenated solvent system of CF containing 1 vol% DIO, all APSCs processed with an XY solvent system had better photovoltaic properties. The use of TN and DPE additives with an optimal boiling point improved the photovoltaic performance significantly. However, the use of either TMB and IN additives with a low boiling point or DBE additive with a very high boiling point resulted in lower photovoltaic performance than that of XY alone. The observed differences in the photovoltaic performance between additives may be attributed to the film morphology and charge lifetime.

## 2. Results and Discussion

**Figure 1:**
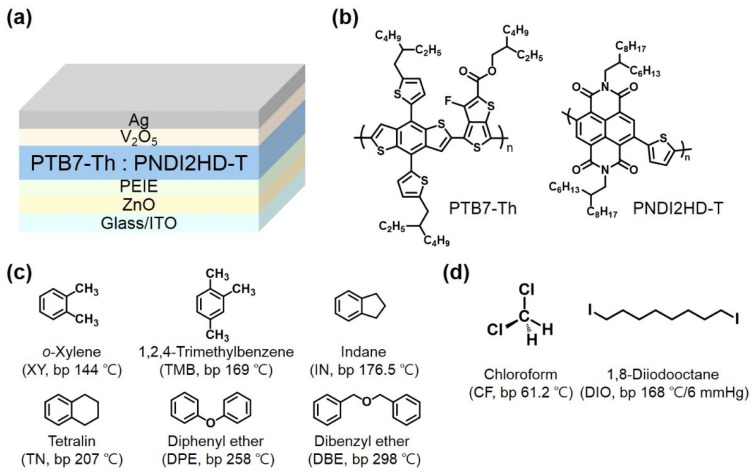
(**a**) Schematic drawing of inverted device structure; (**b**) chemical structure of PTB7-Th (*p*-type polymer) and PNDI2HD-T (*n*-type polymer); (**c**) chemical structure of non-halogenated solvents used in this study; (**d**) chemical structure of halogenated solvents used in this study.

To investigate the correlation between photovoltaic properties and the types of non-halogenated additives, we fabricated inverted-type APSCs with a structure consisting of an ITO/ZnO/PEIE/active layer/V_2_O_5_/Ag with active layers comprising PTB7-Th:PNDI2HD-T blend films spin-coated from an XY solution containing an optimal concentration of an additive (TMB, IN, TN, DPE, or DBE). In addition, APSCs prepared from CF containing DIO were fabricated for comparison with the APSCs fabricated from non-halogenated solvent systems. Figure 1 shows the inverted photovoltaic device structure and the chemical structures of photoactive polymers and solvents. The PTB7-Th and PNDI2HD-T polymers were synthesized via Stille polymerization and purified with methanol, acetone, hexane, cyclohexane (CH), dichloromethane (DCM), and CF solvents in a Soxhlet extraction process. CH, DCM, and CF batches were collected and used to fabricate APSCs. Each batch of PTB7-Th and PNDI2HD-T was blended to prepare APSCs. The best-performing combinations of PTB7-Th and PNDI2HD-T were the CH (M_n_ = 9400 Da, PDI = 2.7) and DCM (M_n_ = 42,000 Da, PDI = 1.9) batches. Both polymer batches were well-dissolved in XY.

**Figure 2:**
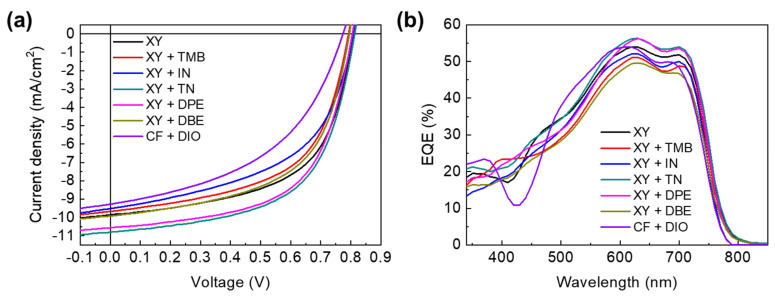
(**a**) *J*−*V* curves and (**b**) EQE spectra of APSCs based on XY and XY + additives. The results from the CF + DIO solvent system are included for comparison.

Figure 2 shows the current density–voltage (*J*−*V*) curves and external quantum efficiency (EQE) spectra of PTB7-Th:PNDI2HD-T blend-film-based solar cells. Table 1 summarizes their photovoltaic properties. Devices fabricated with the halogenated solvent system of CF + DIO (1 vol%) exhibited an average PCE of 3.17%. Devices with XY only exhibited a higher (by 40%) average PCE of 4.44% compared with that of CF + DIO devices. To further improve XY-based APSC performance, we added non-halogenated additives (TMB, IN, TN, DPE, and DBE) to XY solutions. In the screening process, the optimal concentrations of the additives were as follows: 3 vol% TMB, 3 vol% IN, 11 vol% TN, 5 vol% DPE, and 0.4 vol% DBE; the average PCEs of APSCs fabricated with these additives were 4.27, 3.74, 5.04, 4.84, and 4.40%, respectively. Notably, not all of the additives had a positive effect on photovoltaic properties; TN and DPE significantly improved the photovoltaic performance (14% and 13% improvement in the PCE, respectively, compared with the PCE of devices with XY only), whereas TMB, IN, and DBE decreased the photovoltaic performance. PCE improvements were mainly attributed to increased short-circuit current (*J*_sc_) values for devices with both TN and DPE. Devices with TN and DPE exhibited enhanced average *J*_sc_ values of 10.71 and 10.48 mA/cm^2^, respectively, whereas devices with TMB, IN, and DBE exhibited decreased *J*_sc_ values of 9.57, 9.28, and 9.78 mA/cm^2^, respectively. EQE spectral shapes were highly similar; however, there were differences in EQE values among APSCs prepared from XY and XY + additive solutions, and current densities estimated from the EQE spectra were in agreement with the *J*_sc_ values.

**Figure 3:**
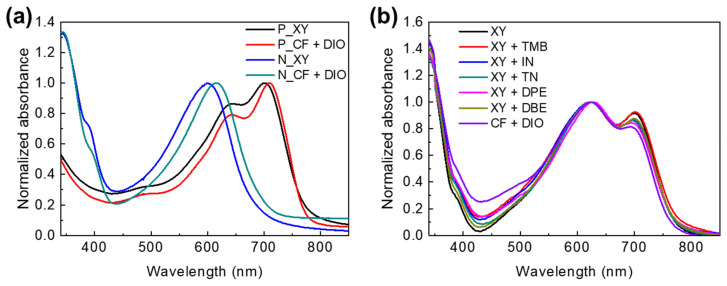
UV−visible absorption spectra of (**a**) PTB7-Th (P) and PNDI2HD-T (N) polymer films processed from XY only and CF + DIO solutions and (**b**) polymer blend films processed from XY, XY + additive, and CF + DIO solutions.

To investigate the differences in APSC photovoltaic properties in terms of the solvents and additives, we performed various measurements and analyses of the light absorption, charge lifetime, mobility, crystallinity, and morphology. Figure 3a shows the normalized UV−visible absorption spectra of PTB7-Th and PNDI2HD-T polymer films processed from XY and CF + DIO solutions, and Figure 3b shows the normalized UV−visible absorption spectra of PTB7-Th:PNDI2HD-T blend films processed from XY, XY + additive, and CF + DIO solutions. PTB7-Th exhibited two major absorption peaks (around 640 nm and 700 nm), and PNDI2HD-T exhibited a broad absorption peak (around 600 nm). XY-based PTB7-Th films had a broader absorption and a weaker vibronic peak. The absorption peaks of both XY-based polymer films were slightly blue-shifted compared with those of the corresponding CF + DIO-based films. This observation indicates that CF + DIO solutions induced higher interchain interactions among the polymer chains. For the blend films, UV−visible absorption spectral features appeared similar for all of the solvent systems and showed two major peaks (around 628 nm and 700 nm). A comparison of the spectra of the blend films with the spectra of the pure polymer films demonstrated that the 700 nm peak of the blend films mainly corresponded to PTB7-Th, and the 628 nm peak of the blend films resulted from the combined absorption of the PTB7-Th and PNDI2HD-T polymers. It may be worthwhile to carefully examine the absorption features in the 450 nm and 700 nm regions simultaneously, considering that the 450 nm region absorption was increased, whereas the 700 nm region absorption was decreased. For instance, XY and XY + additive solutions resulted in an aggregated film morphology (see below) with weak and strong absorptions in the 450 and 700 nm regions, respectively, and the CB + DIO solution resulted in a well-mixed blend morphology with relatively strong and weak absorptions in the 450 and 700 nm regions, respectively.

**Figure 4:**
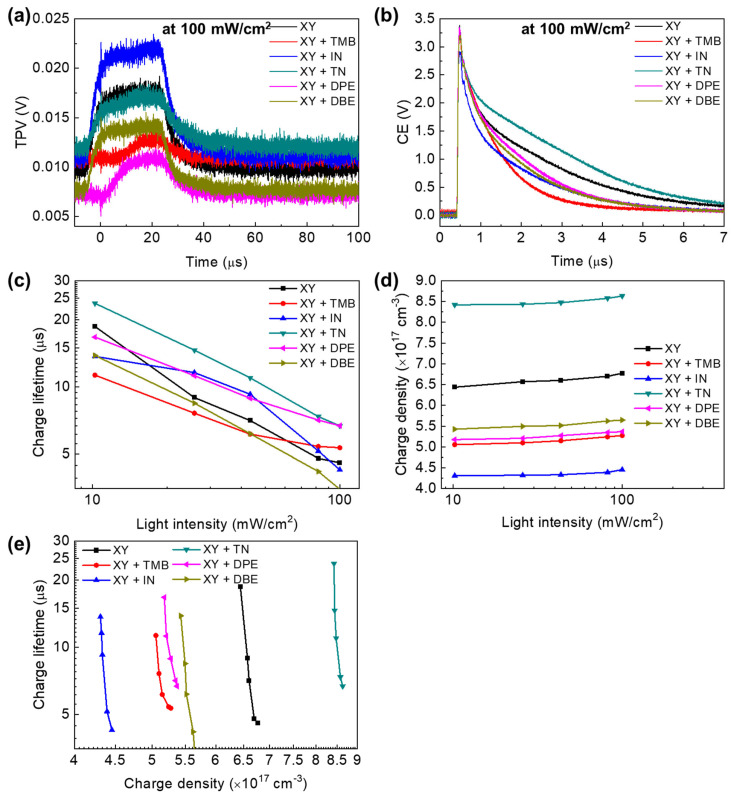
(**a**) TPV profiles measured at 100 mW/cm^2^ with a green LED pulse; (**b**) CE curves measured at 100 mW/cm^2^; (**c**) plot of light intensity versus charge lifetime; (**d**) plot of light intensity versus charge density; (**e**) plot of charge density versus charge lifetime for APSCs depending on the type of processing solution.

Transient photovoltage (TPV) measurements were performed to determine the relationship between charge lifetime and photovoltaic properties. Charge extraction (CE) measurements were also carried out to determine the charge density of the device. TPV and CE measurements were performed at five different light intensities (10, 26, 43, 82, and 100 mW/cm^2^). Figure 4a,b show the TPV and CE profiles of various processing solution-based devices under 100 mW/cm^2^ light illumination. The charge lifetime was obtained by fitting TPV profiles with the exponential function, and the charge density was obtained by integrating CE profiles (see the Supplementary Materials section for the detailed procedure). Figure 4c shows the relationship between light intensity and charge lifetime. As the light intensity decreased, the charge lifetime became longer. This can be explained by the reduced recombination with decreasing light intensity and the lower number of photo-generated carriers. Figure 4d shows that the charge density increased as the light intensity increased. Figure 4e shows the plots of charge density and charge lifetime. All of the devices exhibited a rapid decrease in charge lifetime with a small charge density increase. In addition, for a given charge lifetime, the order of the charge density was consistent with that of the PCE (except for the XY + DPE-based device), and it was strongly correlated with the PTB7-Th:PNDI2HD-T blend morphology. If the charge lifetimes were extrapolated, the charge lifetime at the same charge density varied over several orders of magnitude. This observation suggests that PTB7-Th:PNDI2HD-T blend films with different morphologies can exhibit substantially different recombination rate constants. As shown in Table 2, the charge density at 100 mW/cm^2^ was 8.63 × 10^17^ cm^−3^ for XY + TN, which was the highest charge density, whereas that of XY, XY + TMB, XY + IN, XY + DPE, and XY + DBE was 6.77, 5.27, 4.45, 5.37, and 5.65 × 10^17^ cm^−3^, respectively. However, the charge density was not simply correlated with *J*_sc_ or PCE. On the other hand, charge lifetime results demonstrated a good correlation. Charge lifetimes were long for high-performance XY + TN- and XY + DPE-based devices, i.e., 6.69 μs. The charge lifetimes for poorly performing XY-, XY + TMB-, XY + IN-, and XY + DBE-based devices were 4.57, 5.33, 4.25, and 3.48 μs, respectively. The charge lifetimes of XY + TN- and XY + DPE-based devices were the longest; thus, the chance of charge extraction without recombination was higher, which explains the high *J*_sc_ values (as well as the high fill factors (FF)) of XY + TN- and XY + DPE-based devices. On the other hand, although charge lifetimes were longer for XY + TMB- and XY + IN-based devices than for XY- and XY + DBE-based devices, *J*_sc_ values were higher for XY- and XY + DBE-based devices than for XY + TMB- and XY + IN-based devices because charge densities were lower for XY + TMB- and XY + IN-based devices than for XY- and XY + DBE-based devices. Specifically, the *J*_sc_ of the XY + IN-based device was low because of the low charge density and the short charge lifetime.

**Figure 5:**
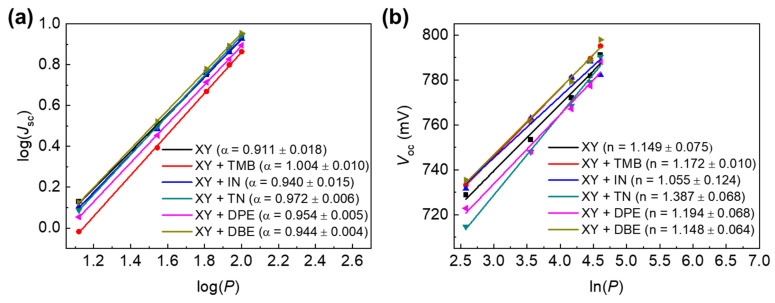
(**a**) *J*_sc_−*P* and (**b**) *V*_oc_−*P* plots of APSCs.

We measured *J*_sc_ and *V*_oc_ according to the light intensity (*P*) to determine the charge recombination mechanism. Light intensity was controlled using neutral-density optical filters, and measurements were performed at five different light intensities (from 13 to 100 mW/cm^2^). The dependence of *J*_sc_ on *P* follows the formula *J*_sc_ ∝ *P*^α^, and bimolecular recombination rarely occurs, as α is close to 1 [37]. Figure 5a shows the linear relationship between the logarithm of *J*_sc_ and the logarithm of *P*. The values of slope α were 0.911, 1.004, 0.940, 0.972, 0.954, and 0.944 for XY, XY + TMB, XY + IN, XY + TN, XY + DPE, and XY + DBE, respectively. The results indicated that the use of additives can help to reduce bimolecular recombination under short-circuit conditions. Figure 5b shows the linear relationship between *V*_oc_ and the natural logarithm of *P* following the formula *V*_oc_ = n(kT/q)ln(*P*). The slope n(kT/q) values were 1.15, 1.17, 1.06, 1.39, 1.19, and 1.15 kT/q for XY, XY + TMB, XY + IN, XY + TN, XY + DPE, and XY + DBE, respectively. The n values of the devices were close to 1, indicating that bimolecular recombination was dominant, with a weak contribution of monomolecular recombination under *V*_oc_ conditions [38]. All of the n values appeared to be similar and close to 1. Therefore, bimolecular recombination was the main recombination pathway near the *V*_oc_ region for all of the APSCs investigated in this study.

**Figure 6:**
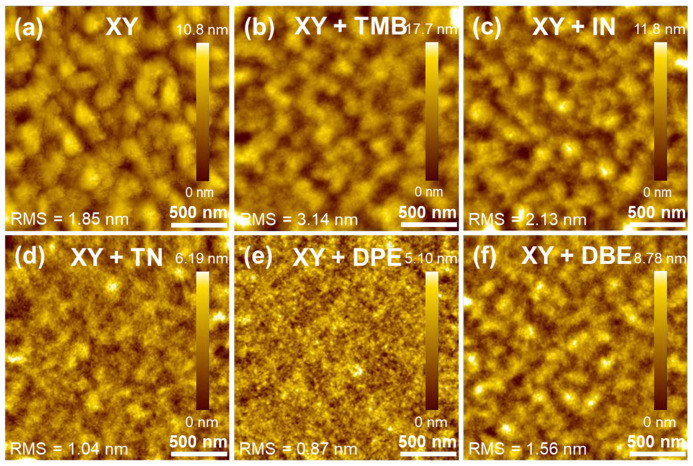
AFM topography images of (**a**) XY-, (**b**) XY + TMB-, (**c**) XY + IN-, (**d**) XY + TN-, (**e**) XY + DPE-, and (**f**) XY + DBE-based blend films.

The morphologies of the polymer blend films were examined by atomic force microscopy (AFM) and transmission electron microscopy (TEM). Figure 6 shows the AFM topography images of PTB7-Th:PNDI2HD-T blend films spin-coated with six different solutions. Among the XY-based films, those with DPE were the smoothest and had the smallest domain size. The root mean square (RMS) values of surface roughness were 1.85, 3.14, 2.13, 1.04, 0.87, and 1.56 nm for XY-, XY + TMB-, XY + IN-, XY + TN-, XY + DPE-, and XY + DBE-based films, respectively. The results were well correlated with the photovoltaic performance; high-performance XY + TN- and XY + DPE-based films were smooth, whereas poorly performing XY + TMB- and XY + IN-based films were rough.

**Figure 7:**
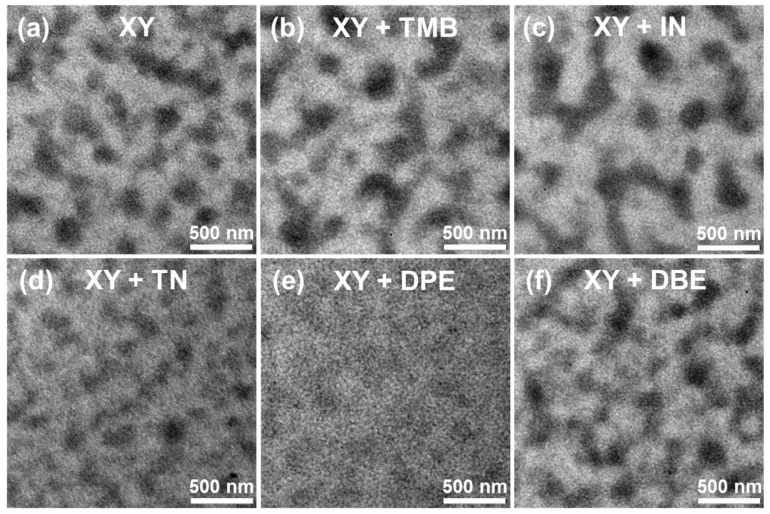
TEM images of (**a**) XY-, (**b**) XY + TMB-, (**c**) XY + IN-, (**d**) XY + TN-, (**e**) XY + DPE-, and (**f**) XY + DBE-based blend films.

Figure 7 shows the TEM images of PTB7-Th:PNDI2HD-T blend films. The features of TEM images were similar to those of AFM topography images. XY-, XY + TMB-, XY + IN-, and XY + DBE-based films showed a largely aggregated phase. In contrast, XY + TN- and XY + DPE-based films had much smaller domain sizes, which are desirable for generating photo-induced carriers.

**Figure 8:**
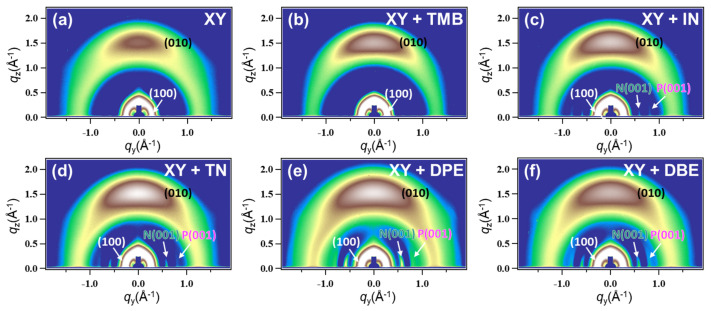
GIXD images of (**a**) XY-, (**b**) XY + TMB-, (**c**) XY + IN-, (**d**) XY + TN-, (**e**) XY + DPE-, and (**f**) XY + DBE-based blend films. The (100) and (010) peaks consist of both *p*- and *n*-type polymers; N(001) corresponds to the (001) peak of *n*-type polymer and P(001) corresponds to the (001) peak of *p*-type polymer.

Two-dimensional grazing incidence X-ray diffraction (GIXD) experiments were conducted to examine the crystallinity and chain orientation of polymer chains in the polymer blend films. Figure 8 shows the GIXD images of PTB7-Th:PNDI2HD-T blend films processed from XY and XY + additive solutions. All of the blend films showed strong (100) peaks in the in-plane direction and π–π stacking peaks in the out-of-plane direction. XY-, XY + TMB-, and XY + IN-based films exhibited weak crystalline features for PNDI2HD-T in the in-plane direction, whereas XY + TN-, XY + DPE-, and XY + DBE-based films showed stronger crystalline features. This observation may be attributed to the film drying speed, considering that XY, TMB, and IN have boiling points below 200 °C, whereas TN, DPE, and DBE have boiling points above 200 °C.

**Figure 9:**
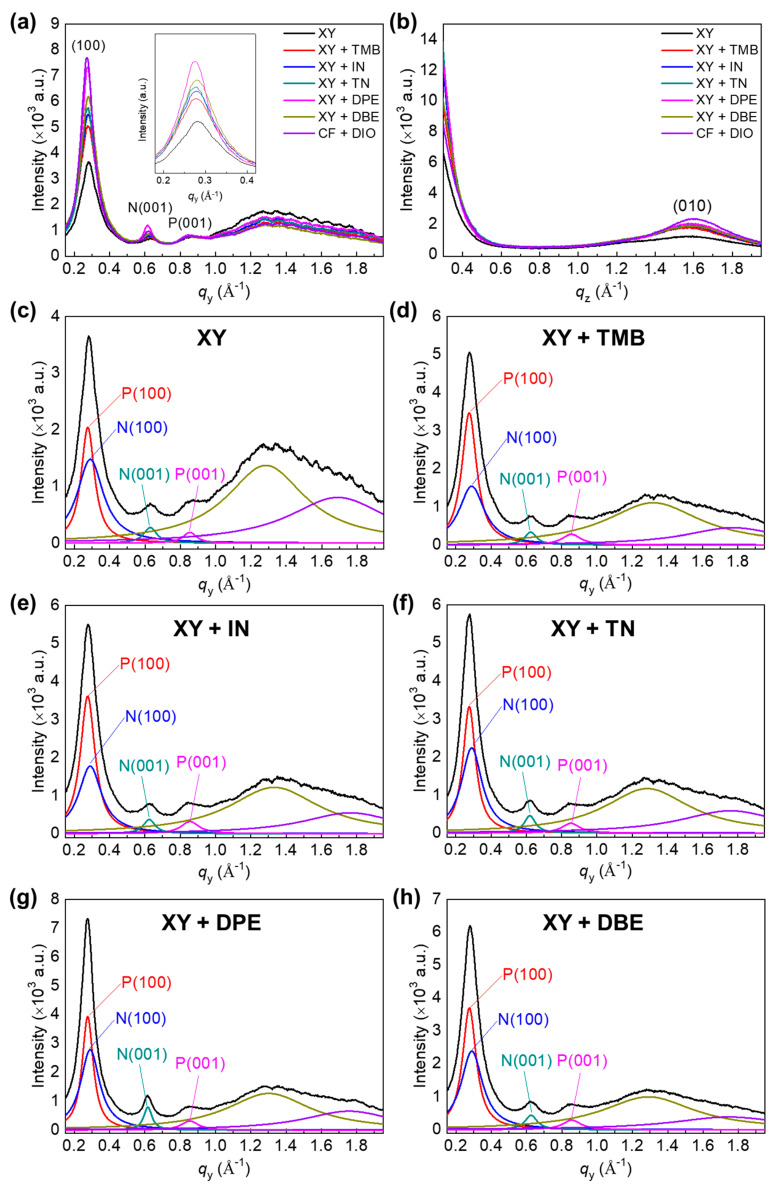
(**a**) In-plane line cuts and (**b**) out-of-plane line cuts of GIXD images in the blend films. Each in-plane line cut with fitted lines of (**c**) XY-, (**d**) XY + TMB-, (**e**) XY + IN-, (**f**) XY + TN-, (**g**) XY + DPE-, and (**h**) XY + DBE-based blend films. Raw data were fitted with the Lorentzian function. P and N indicate the PTB7-Th and PNDI2HD-T polymers, respectively.

For detailed analysis, the in-plane line cut spectra of PTB7-Th:PNDI2HD-T blend films were extracted (Figure 9). The peaks corresponding to the (100) plane of PTB7-Th, the (100) plane of PNDI2HD-T, the (001) plane of PNDI2HD-T, and the (001) plane of PTB7-Th were observed at around 0.27, 0.29, 0.62, and 0.86 Å^−1^, respectively. Table S1 summarizes the *d*-spacing value and correlation length (*L*_c_) of each peak and contains information for the PTB7-Th and PNDI2HD-T polymer films processed from the XY solvent only. The *L*_c_ values in the (100) plane of *p*-type polymers in the blends were increased in the order of XY + TMB, XY + IN, XY + DBE, XY, XY + TN, and XY + DPE (11.0, 11.2, 12.0, 12.3, 13.0, and 14.8 nm, respectively). The *L*_c_s in the (100) plane of *n*-type polymers were lower than those of the corresponding *p*-type polymers and were increased in the order of XY, XY + TMB, XY + IN, XY + DBE, XY + TN, and XY + DPE (6.3, 6.5, 7.2, 7.6, 7.9, and 8.5 nm, respectively). Overall, high-boiling-point additives contributed to larger *L*_c_s because of the slow drying time, which provided sufficient time for the self-organization of polymer chains. An exception was that XY + DBE resulted in a medium *L*_c_, which might be explained by the use of a relatively smaller amount of the DBE additive (0.4 vol%). More importantly, *L*_c_ analysis demonstrated that PTB7-Th polymer chains tend to form more and longer crystalline domains compared with those of PNDI2HD-T polymer chains; the fibrous features seen in AFM and TEM images were mainly attributed to PTB7-Th polymers. However, it should be noted that the roughness and domain sizes of the blend films shown in AFM and TEM images were not in agreement with the *L*_c_ in the (100) plane of the PTB7-Th polymer; compared with XY + TMB- and XY + IN-based films with rough and large domains, XY + TN- and XY + DPE-based films with smooth and small domains had longer *L*_c_s in the (100) planes of PTB7-Th and PNDI2HD-T polymers. This finding suggests that the large domains of blend films processed with XY + TMB and XY + IN may be composed of PTB7-Th and PNDI2HD-T polymers in a simply aggregated amorphous phase. In contrast, for XY + TN- and XY + DPE-based films, the TN and DPE additives could help each type of polymer chain to segregate into separate crystalline phases to achieve favorable effects, i.e., smoother and more crystalline blend films.

**Figure 10:**
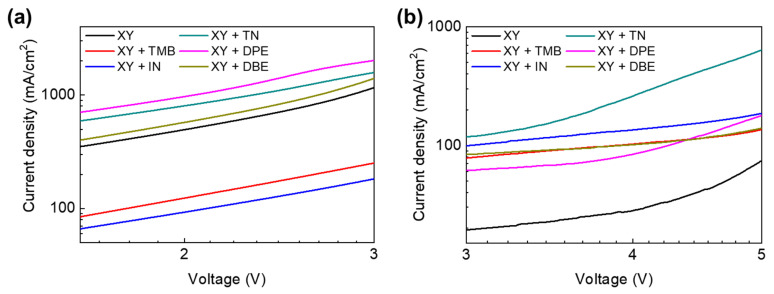
*J*−*V* characteristics of the SCLC regime of (**a**) hole-only devices and (**b**) electron-only devices.

Hole and electron mobilities were measured using hole-only and electron-only devices with the respective device structures of ITO/PEDOT:PSS/PTB7-Th:PNDI2HD-T/MoO_3_/Al and ITO/PEIE/PTB7-Th:PNDI2HD-T/CsCO_3_/Al. The hole and electron mobilities of PTB7-Th:PNDI2HD-T blend films were calculated by fitting *J*−*V* curves based on the space charge-limited current (SCLC) model. Figure 10 shows the SCLC region of the *J*−*V* curves of PTB7-Th:PNDI2HD-T blend films spin-coated from XY and XY + additive solutions. Table 3 summarizes the hole and electron mobilities and their ratios. Hole mobilities ranged from 10^−5^ to 10^−4^ cmV^−1^s^−1^, and electron mobilities ranged from 10^−6^ to 10^−5^ cmV^−1^s^−1^. The higher hole mobilities compared with the electron mobilities indicated the higher self-organizing tendency of the PTB7-Th polymer, as demonstrated by the GIXD data. The low electron mobility values may be attributed to the low crystallinity and shorter *L*_c_ of the PNDI2HD-T polymer, which could be one of the main factors contributing to the low FF values of the APSCs.

**Figure 11:**
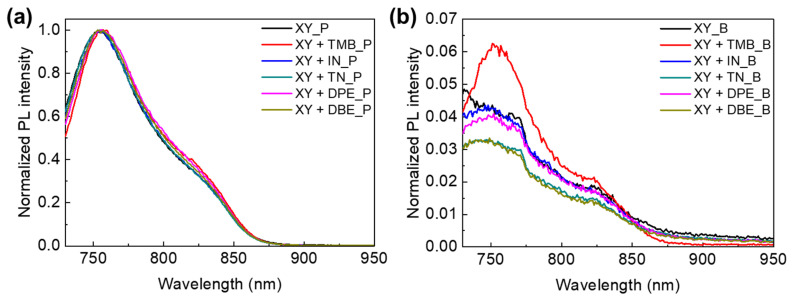
Photoluminescence spectra for (**a**) *p*-type polymer films and (**b**) blend polymer films.

Photoluminescence (PL) spectra were obtained to investigate the exciton separation effect according to the PTB7-Th:PNDI2HD-T blend films processed from XY and XY + additive solutions. The PTB7-Th and PTB7-Th:PNDI2HD-T blend films were irradiated with 630 nm excitation light. The PL quenching efficiency was calculated by dividing the integral area of (*P*-*B*) by the integral area of *P*, where *P* is the PL spectrum of the PTB7-Th film, and *B* is the PL spectrum of the PTB7-Th:PNDI2HD-T blend film. Figure 11 shows the normalized PL spectra of PTB7-Th films and PTB7-Th:PNDI2HD-T blend films processed with six different solutions, and Table S2 presents the data for the PL quenching efficiency. The PL spectra of PTB7-Th polymer films and the blend polymer films were normalized to the highest intensity in the raw PL spectra of PTB7-Th polymer films. The estimated PL quenching efficiencies were almost 95% for all of the blend films. The results indicated that excitons were well split into holes and electrons in the blend films for all of the processing solvent systems, suggesting that the charge lifetime may be more important than exciton splitting for the APSCs.

**Figure 12:**
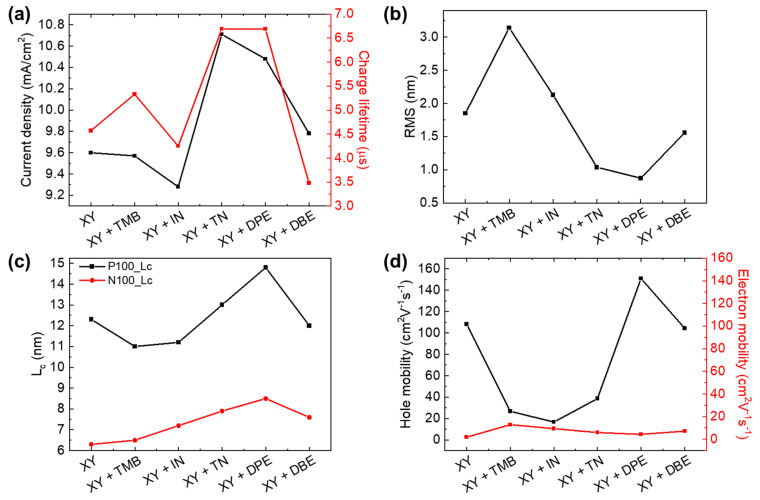
Correlations between (**a**) *J*_sc_ and charge lifetime, (**b**) RMS, (**c**) *L*_c_ in the (100) plane of each polymer, and (**d**) hole and electron mobilities.

The photovoltaic properties of PTB7-Th:PNDI2HD-T-based APSCs fabricated using the non-halogenated solvent XY with five additives were investigated in this study. The key results are summarized in Figure 12, which shows the correlations among photovoltaic properties, charge lifetime, morphology, charge mobility, and solvent systems. The PCEs of APSCs with non-halogenated additives were mainly dependent on the *J*_sc_ of each APSC. A good correlation between *J*_sc_ and charge lifetime was observed, as shown in Figure 12a, demonstrating that the charge lifetime is critical for high photovoltaic performance. The morphology and surface roughness of polymer blend films were affected by the type of non-halogenated additive. The lower the surface roughness, the longer the *L*_c_, as shown in Figure 12b,c. Additionally, the hole mobility exhibited a strong correlation with the crystallinity and *L*_c_ of *p*-type PTB7-Th polymer domains, as shown in Figure 12d; however, increasing the hole mobility using TN or DPE additives did not lead to increased PCE, suggesting that the carrier mobility is not a critical factor in PTB7-Th:PNDI2HD-T-based APSCs. The results of this study suggest that the use of a relevant non-halogenated additive can be an effective strategy in improving the photovoltaic performance in APSCs by increasing charge lifetime (resulting in improved *J*_sc_ and PCE values), as well as in reducing the phase separation between *p*- and *n*-type polymers to an optimal size [27].

**Figure 13:**
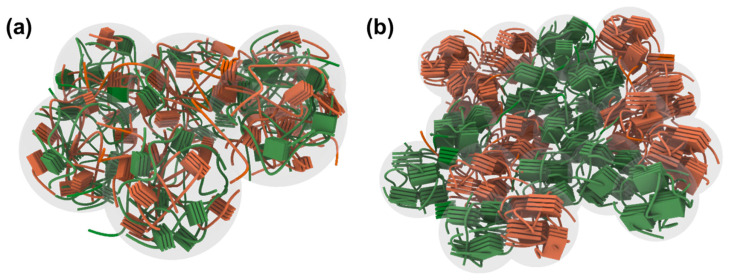
Schematic drawing of (**a**) randomly aggregated polymer blends representing the blend films processed from XY, XY + TMB, and XY + IN solutions and (**b**) polymer blends with internetworked *p*-type polymers representing the blend films processed from XY + TN and XY + DPE solutions. *p*-type and *n*-type polymers are presented by green and orange colors, respectively, and the aggregated domains are presented by light gray circles.

Based on these results, the polymer blend morphologies are illustrated in Figure 13, showing randomly aggregated polymer blends and polymer blends with internetworked PTB7-Th polymers. Figure 13a shows aggregates of the amorphous *p*-type polymers (green) and aggregates of *n*-type polymers (orange). These aggregates were large and rough but did not have a long-range order of constituted polymer chains. On the other hand, Figure 13b shows interconnected domains formed by the crystalline *p*-type polymers, which offer an efficient hole-transporting pathway. In addition, the less crystalline *n*-type polymers were not well aligned. As a result, the *L*_c_ in the (100) plane of *p*-type polymers was longer than that of *n*-type polymers, and the hole mobility was faster. The morphology of blend films processed with XY + TMB and XY + IN was similar to that shown in Figure 13a, and the morphology of blend films processed with XY + TN and XY + DPE was similar to that shown in Figure 13b. The APSCs processed with XY + TN and XY + DPE exhibited high PCEs of 5.17% and 5.01%, respectively, because the interconnected nanoscale crystalline domains of *p*-type polymers helped holes move faster. In contrast, APSCs processed with XY + TMB and XY + IN yielded lower PCEs of 4.35% and 4.01%, respectively, due to the amorphous aggregated domains, where carrier transport is not efficient and charge lifetime is short. Overall, the large and rough domains of the all-polymer blend films were composed of mixed amorphous polymer blends, and the small and smooth domains of the films were composed of nanoscale, phase-separated, long-range-ordered crystalline polymers; the latter is important to improve photovoltaic properties, and the use of an appropriate additive could be an effective strategy to achieve this.

## 3. Conclusions

In this study, we investigated the effect of non-halogenated additives on the photovoltaic properties of PTB7-Th:PNDI2HD-T-based APSCs by characterizing PTB7-Th:PNDI2HD-T blend films. XY was the main solvent, and TMB, IN, TN, DPE, and DBE were used as non-halogenated additives. APSCs processed with CF + DIO were compared with APSCs processed with non-halogenated solvent systems (XY and XY + additive). PCEs were increased in the order of CF + DIO, XY + IN, XY + TMB, XY + DBE, XY, XY + DPE, and XY + TN. The determining parameter affecting the PCE was *J*_sc_. TPV measurements revealed that devices with higher *J*_sc_ values had a longer charge lifetime; APSCs processed with XY + TN and XY + DPE had the longest charge lifetime. Analyses of the blend morphology by AFM, TEM, GIXD, and charge mobility measurements revealed good correlations among the polymer domain size, crystallinity, and charge mobility. The hole mobility was higher, as the *L*_c_ in the (100) plane of the PTB7-Th polymer was longer. A nanoscale fibrous network of crystalline polymer domains was formed when favorable non-halogenated additives were used, whereas large domains composed of randomly aggregated polymers were formed when unfavorable solvent systems were used. In conclusion, APSCs processed with favorable non-halogenated additives had small crystalline domains with a high charge lifetime and mobility, resulting in high PCEs. This study demonstrates the critical role of non-halogenated additives in modulating the polymer blend morphology, as well as the correlations among morphology, charge lifetime, and photovoltaic performance.

## Figures and Tables

**Table 1:** Photovoltaic parameters of APSCs prepared from XY, XY + additives, and CF + DIO solutions.

Solvent	*V*_oc_ (V)	*J*_sc_ (mA/cm^2^)	FF	PCE (%) *^a^*
XY	0.80 ± 0.01 (0.81)	9.60 ± 0.16 (9.86)	0.579 ± 0.05 (0.59)	4.44 ± 0.14 (4.66)
XY + TMB	0.793 ± 0.001 (0.79)	9.57 ± 0.11 (9.67)	0.563 ± 0.003 (0.57)	4.27 ± 0.06 (4.35)
XY + IN	0.80 ± 0.01 (0.81)	9.28 ± 0.15 (9.52)	0.51 ± 0.01 (0.52)	3.74 ± 0.18 (4.01)
XY + TN	0.81 ± 0.01 (0.81)	10.71 ± 0.08 (10.80)	0.58 ± 0.01 (0.59)	5.04 ± 0.10 (5.17)
XY + DPE	0.797 ± 0.001 (0.80)	10.48 ± 0.13 (10.56)	0.597 ± 0.003 (0.60)	4.84 ± 0.11 (5.01)
XY + DBE	0.80 ± 0.01 (0.79)	9.78 ± 0.15 (9.93)	0.57 ± 0.01 (0.57)	4.40 ± 0.07 (4.49)
CF + DIO	0.77 ± 0.01 (0.77)	9.03 ± 0.24 (9.27)	0.45 ± 0.01 (0.47)	3.17 ± 0.18 (3.37)

***^a^*** The average values were calculated from eight devices.

**Table 2:** Summary of charge density and charge lifetime depending on solvent system and light intensity.

		Light Intensity (mW/cm^2^)				
		100	82	43	26	10
XY	Charge density (×10^17^ cm^−3^)	6.77	6.70	6.60	6.56	6.44
	Lifetime (μs)	4.57	4.78	7.06	8.95	18.68
XY + TMB	Charge density (×10^17^ cm^−3^)	5.27	5.24	5.15	5.10	5.05
	Lifetime (μs)	5.33	5.40	6.14	7.62	11.29
XY + IN	Charge density (×10^17^ cm^−3^)	4.45	4.40	4.33	4.32	4.31
	Lifetime (μs)	4.25	5.15	9.27	11.60	13.71
XY + TN	Charge density (×10^17^ cm^−3^)	8.63	8.58	8.47	8.43	8.42
	Lifetime (μs)	6.69	7.36	10.98	14.63	23.76
XY + DPE	Charge density (×10^17^ cm^−3^)	5.37	5.35	5.27	5.21	5.18
	Lifetime (μs)	6.69	7.09	8.89	11.23	16.75
XY + DBE	Charge density (×10^17^ cm^−3^)	5.65	5.62	5.51	5.49	5.43
	Lifetime (μs)	3.48	4.18	6.16	8.45	13.84

**Table 3:** Summary of hole mobility (μ_h_)_,_ electron mobility (μ_e_), and the ratio of hole and electron mobilities (μ_h_/μ_e_) of XY- and XY + additive-based APSCs.

Solvent	μ_h_ (cm^2^·V^−1^·s^−1^)	μ_e_ (cm^2^·V^−1^·s^−1^)	μ_h_/μ_e_
XY	1.08 (±0.11) × 10^−4^	1.87 (±1.1) × 10^−6^	57.7
XY + TMB	2.68 (±0.98) × 10^−5^	1.29 (±0.88) × 10^−5^	2.08
XY + IN	1.67 (±0.73) × 10^−5^	9.40 (±2.5) × 10^−6^	1.77
XY + TN	3.86 (±0.53) × 10^−5^	5.95 (±1.1) × 10^−6^	6.49
XY + DPE	1.51 (±0.16) × 10^−4^	4.35 (±0.88) × 10^−6^	34.7
XY + DBE	1.04 (±0.13) × 10^−4^	7.18 (±3.1) × 10^−6^	14.5

## Data Availability

All experimental and supporting data are provided in the Supplementary Materials.

## Supplementary Materials

The following supporting information can be downloaded at https://www.mdpi.com/article/10.3390/polym15061354/s1: Experimental details (polymer synthesis and characterization methods); Table S1. Summary of GIXD parameters in the polymer films; Table S2. PL quenching efficiencies of XY- and XY + additive-based polymer films. Reference [39] are cited in the Supplementary Materials.
