# Supplementary material for: Critical Role of Non-Halogenated Solvent Additives in Eco-Friendly and Efficient All-Polymer Solar Cells
Source: Polymers (Basel). 2023 Mar 8;15(6):1354. doi: 10.3390/polym15061354 (PMC10056264; doi:10.3390/polym15061354)
Supplement: Supplementary file 1 [file polymers-15-01354-s001.zip › polymers-2220716-supplementary.pdf]

Supporting Information

Critical Role of Non-halogenated Solvent Additives in Eco-friendly and Efficient All-Polymer Solar Cells

Saeah Kim,[1] Huijeong Choi,[2] Myeongjae Lee,[3] Hyeseung Jung,[1] Yukyung Shin,[1] Seul Lee,[2] Kyungkon Kim,[1] Myung Hwa Kim,[1] Kyungwon Kwak,[3] and BongSoo Kim[2,4,5,*]

[1] Department of Chemistry & Nano Science, Ewha University, Seoul 03760, Republic of Korea
[2] Department of Chemistry, Ulsan National Institute of Science and Technology (UNIST), Ulsan 44919, Republic of Korea
[3] Department of Chemistry, Korea University, Seoul 02841, Republic of Korea
[4] Graduate School of Semiconductor Materials and Device Engineering, Ulsan National Institute of Science and Technology (UNIST), Ulsan 44919, Republic of Korea
[5] Graduate School of Carbon Neutrality, Ulsan National Institute of Science and Technology (UNIST), Ulsan 44919, Republic of Korea
* **Correspondence:** bongsoo@unist.ac.kr; Tel.: +82-52-217-3197; Fax: +82-52-217-2279

Experimental Section

Materials

The solvent *o*-xylene (XY) and chloroform (CF) were purchased from Sigma-Aldrich (St. Louis, MO, USA). 1,2,4-Trimethylbenzene (TMB) and tetralin (TN) were purchased from Kanto chemical Co., Inc. (Tokyo, Japan). Indane (IN) and diphenyl ether (DPE) were purchased from Tokyo Chemical Industry Co., Ltd. (Tokyo, Japan). Dibenzyl ether (DBE) and 1,8-diiodooctane (DIO) were purchased from Alfa Aesar (Haverhill, MS, USA). Zinc acetate used as a precursor of ZnO solution and polyethylenimine (PEIE) were purchased from Sigma-Aldrich (St. Louis, MO, USA). Vanadium pentaoxide (V_2O_5) used as a hole transport layer (HTL) and Ag wire used as an anode were purchased from Sigma-Aldrich (St. Louis, MO, USA) and P&H Tech. (Yongin-si, South Korea), respectively. Anhydrous *N,N*-dimethylformamide (DMF), anhydrous toluene, and tetrakis(triphenylphosphine)palladium(0) ($Pd(PPh_3)_4$) were purchased from Sigma-Aldrich (St. Louis, MO, USA). High grade common solvents were purchased from Daejung Chemical (Siheung-si, Republic of Korea). 4,9-dibromo-2,7-bis(2-hexyldecyl)benzo[lmn][3,8]phenanthroline-1,3,6,8(2H,7H)-tetraone (Br-NDI2HD-Br), (4,8-bis(5-(2-ethylhexyl)thiophen-2-yl)benzo[1,2-*b*:4,5-*b*']dithiophene-2,6-diyl)bis(trimethylstannane) (BDTT-ditin), 2-ethylhexyl 4,6-dibromo-3-fluorothieno[3,4-*b*]thiophene-2-carboxylate (FTT) were purchased from SunaTech Inc (China). 2,5-bis(trimethylstannyl)thiophene was synthesized following reported literature [39]. $CDCl_3$ solvent was purchased from Cambridge Isotope Laboratories (Middlesex County, MS, USA). Toluene and DMF solvents for polymerization were respectively degassed by Freeze-Pump-Thaw three cycling and added to the reaction mixture. The other common solvents were used as received. PTB7-Th and PNDI2HD-T polymer were synthesized via Stille coupling, the details of which are provided below.

Synthesis of poly(2,7-bis(2-hexyldecyl)-4-(thiophen-2-yl)benzo[lmn][3,8]phenanthroline-1,3,6,8(2H,7H)-tetraone), **PNDI2HD-T**: Br-NDI2HD-Br (0.400 g, 0.4583 mmol), 2,5-bis(trimethyltin)-thiophene (0.187 mg, 0.4583 mmol), tetrakis(triphenylphosphine)palladium (0.0212 mg, 4 mol%) were dissolved in DMF (1.5 mL), toluene (7.5 mL) and the solution was heated with stirring at 60 °C. Reaction temperature was raised to 90 °C gradually at a rate of 1 °C/150 s, and gradually at a rate of 1 °C/300 s to 100 °C. After 317 min, 2-bromothiophene (0.1 mL) was added to the solution and then stirred for 1 h. Next, the reaction mixture was diluted with chloroform (50 mL) and added to diethylammonium diethyldithiocarbamate (15 mg) aqueous solution (40 mL) in 250 mL round-bottomed flask

using chloroform (40 mL) and stirred at 50 °C for 1 h. After cooling to room temperature, the solution was extracted with chloroform (60 mL) and the organic solution was washed with water (100 mL, 2 times) and brine (100 mL). The organic solvent was evaporated and then the crude polymer was redissolved in chloroform (15 mL) and then precipitated in methanol (250 mL). The precipitated polymer was collected by filtration and dried. The dried polymer was further purified by Soxhlet extraction using methanol, acetone, hexane, cyclohexane, dichloromethane. The dichloromethane fraction, which was used in this work, was precipitated in methanol (250 mL), filtered, and dried in vacuum to yield polymer **PNDI2HD-T** (0.270 g, 74 % yield). ^{1}H-NMR (300 MHz, $CDCl_3$): δ = 8.974 (s, 2H), 7.460 (s, 2H), 4.2-4.1 (br, 4H), 2.06-2.00 (br, 2H), 1.39-1.29 (br, 20H), 1.26-1.20 (br, 28H), 0.85-0.79 (t, 12H). FT-IR (KBr): 2927 cm^{-1} (C-H), 2847 cm^{-1} (C-H) 1706 cm^{-1} (C=O) 1663 cm^{-1} (C=O). Gel-permeation chromatography (GPC) (*o*-dichlorobenzene, 80 °C) M_n =44,000 Da, M_w = 83,000 Da, PDI = 1.8.

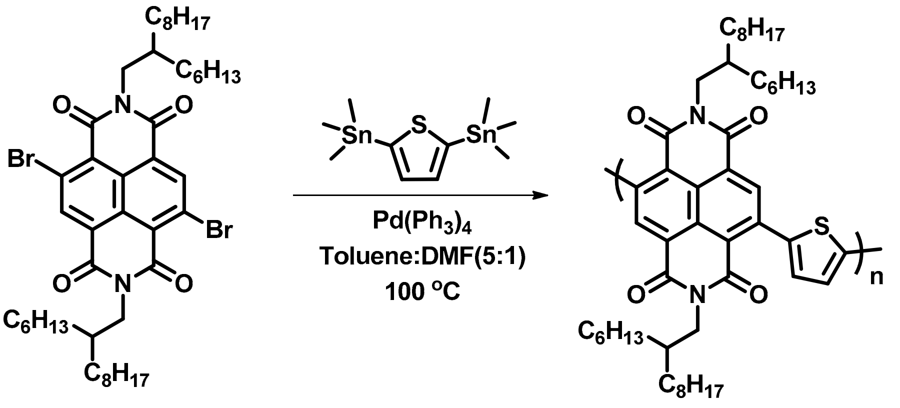

Br-NDI2HD-Br **PNDI2HD-T**

Synthesis of poly(2-ethylhexyl 4-(4,8-bis(5-(2-ethylhexyl)thiophen-2-yl)benzo[1,2-b:4,5-b']dithiophen-2-yl)-3-fluorothieno[3,4-b]thiophene-2-carboxylate), **PTB7-Th:** BDTT-ditin (0.500 g, 0.5527 mmol), FTT (0.261.03 mg, 0.4583 mmol), tetrakis(triphenylphosphine)palladium (0.0255 mg, 4 mol%) were dissolved in DMF (1.83 mL) and toluene (9.17 mL). The solution was heated to 110 °C with stirring. After 145 min, 2-bromothiophene (0.1 mL) was added to the solution, which was then stirred for 1 h. Next, the reaction mixture was diluted with chloroform (50 mL) and added to diethylammonium diethyldithiocarbamate (20 mg) aqueous solution (50 mL) in 250 mL round-bottomed flask and stirred at 50 °C for 1 h. After cooling to room temperature, the solution was extracted with chloroform (70 mL) and the organic solution was washed with water (100 mL, 2 times) and brine (100 mL). The organic solvent was evaporated. The crude polymer was redissolved in chloroform (20 mL) and then was precipitated in methanol (300 mL). The precipitated polymer was collected by filtration and dried. The dried polymer was further purified by Soxhlet extraction using methanol, acetone, hexane, cyclohexane, dichloromethane. The cyclohexane fraction, which was used in this work, was precipitated in methanol (300 mL), filtered, and dried in vacuum to yield polymer **PTB7-Th** (0.460 g, 95.3 % yield). ^{1}H-NMR (300 MHz, $CDCl_3$): δ = 8.15-7.95 (br, 1H), 7.55-7.38 (br, 1H), 7.34-7.28 (br, 1H), 6.95-6.92 (br, 1H), 6.83-6.75 (br, 1H), 4.32-4.28 (br, 6H), 3.02-2.85 (br, 3H), 1.43-1.38 (br,24H), 1.01-0.95 (br, 9H). FT-IR (KBr): 2955 cm^{-1} (C-H), 2921 cm^{-1} (C-H), 2857 cm^{-1} (C-H), 1699 cm^{-1} (C=O). Gel-permeation chromatography (GPC) (*o*-dichlorobenzene, 80 °C) M_n = 9,400 Da, M_w = 26,000 Da, PDI = 2.7.

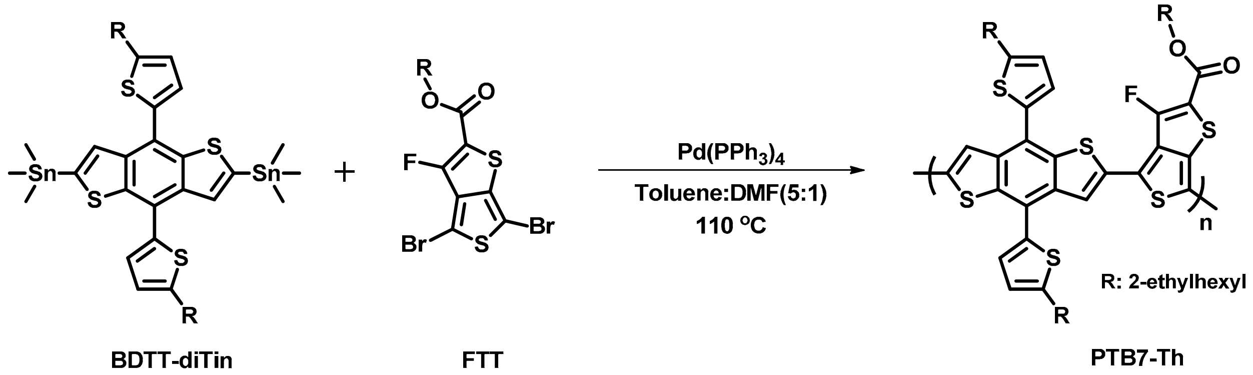

Characterization of Synthesized Polymers:

To identify the molecular structures of all the synthesized products (Figure S1–S5), ^{1}H-NMR spectra were acquired using Bruker Avance III 300MHz with $CDCl_3$ as solvent. The molecular weights and polydispersity index (PDI) of polymers were determined by an Agilent GPC system (GPC 1200 system) at 80 °C. *o*-Dichlorobenzene was used as the eluent, and standard polystyrenes were used for molecular weight calibration.

Cyclic voltammetry (CV) measurement was conducted on an CH instruments electrochemical analyzer. Acetonitrile solution containing 0.1 M tetrabutylammonium hexafluorophosphate (Bu_4NPF_6) was used as the electrolyte solution. A platinum (Pt) wire was used as the working electrode, where polymer films were coated. Another bare Pt wire was used as the counter electrode and an Ag/Ag^+ electrode as a reference electrode. Potential was swept at a rate of 50 mV/s. The potentials were calibrated by the ferrocene/ferrocenium (Fc/Fc^+) oxidation potential (–4.8 eV).

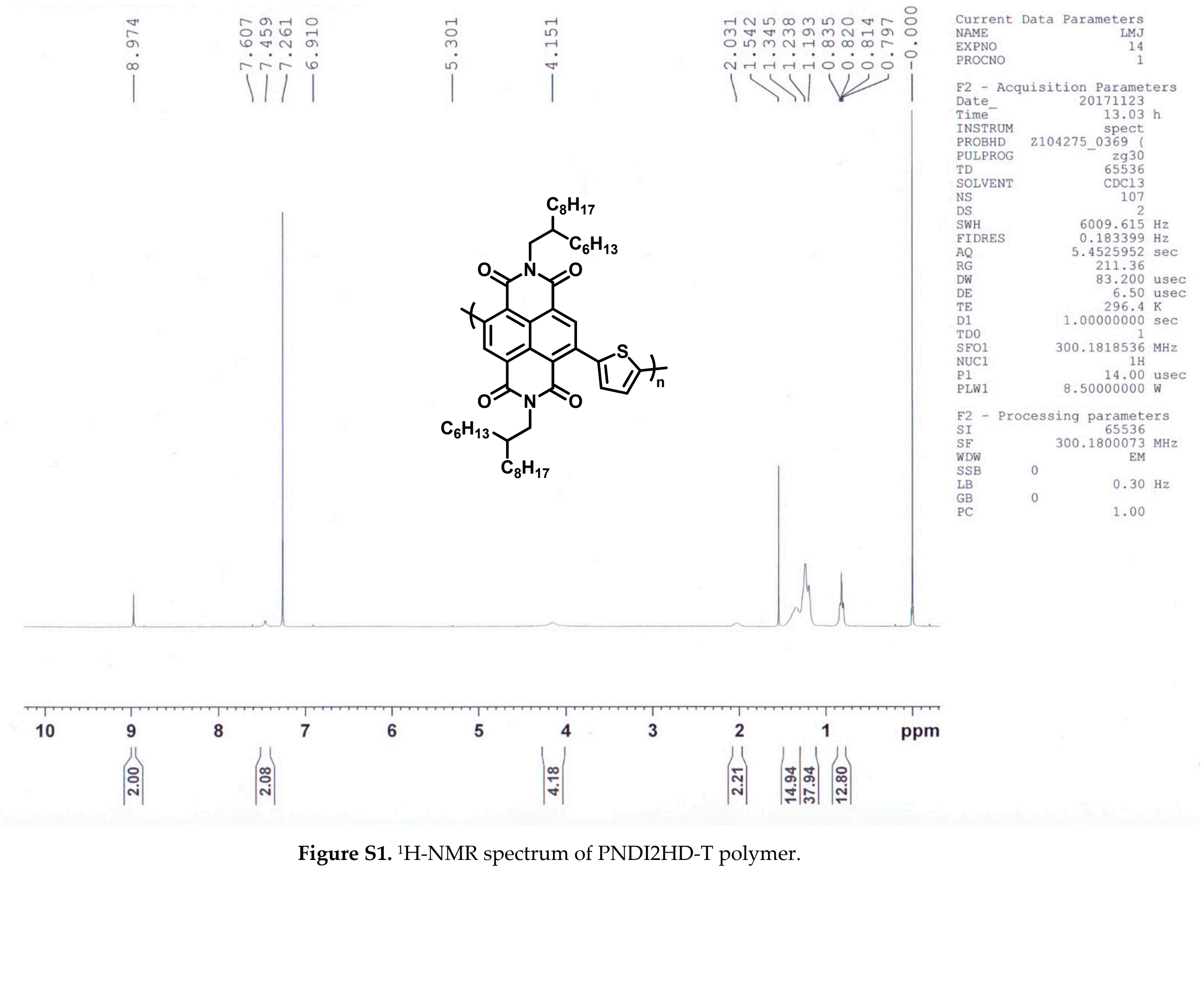

Figure S1. ^{1}H-NMR spectrum of PNDI2HD-T polymer.

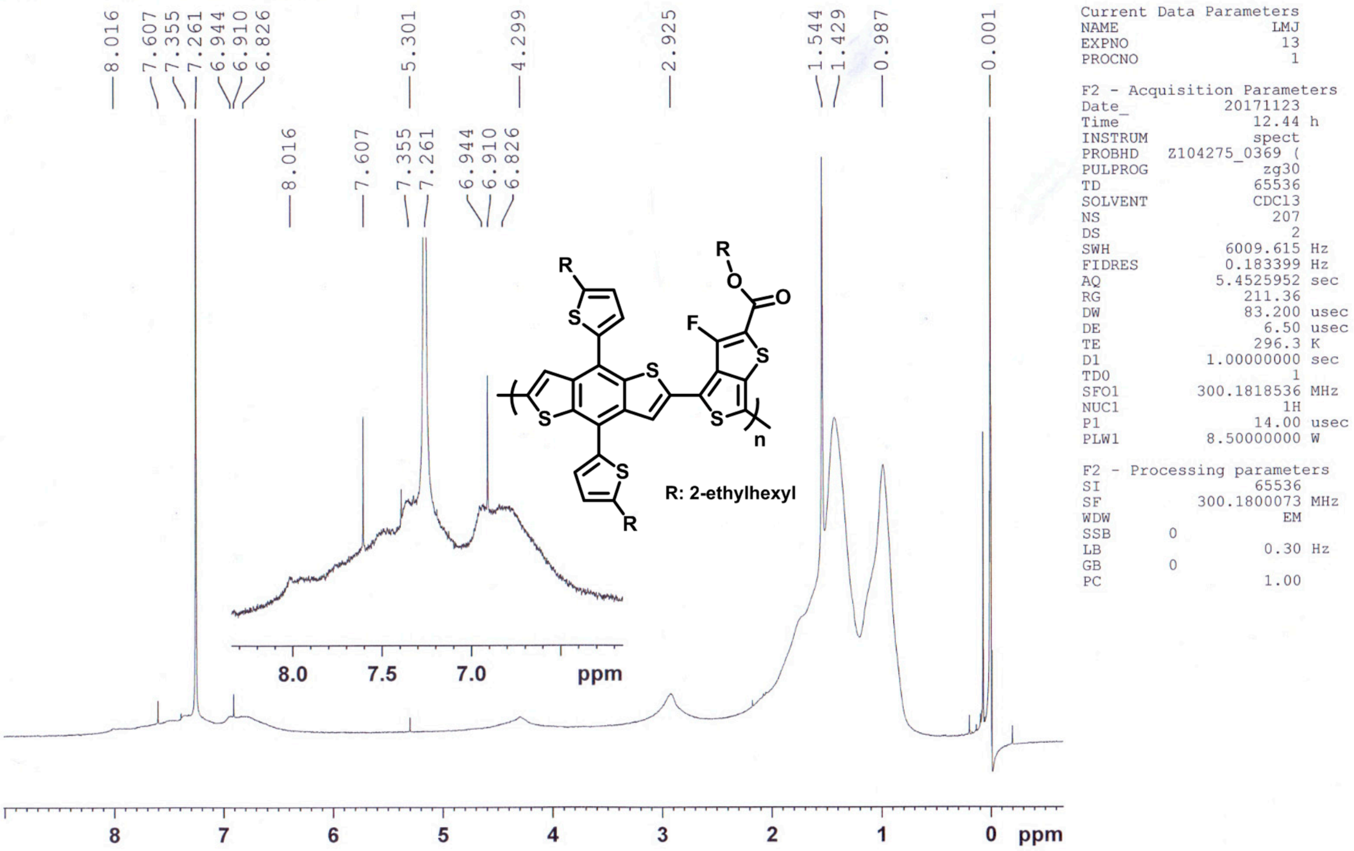

Figure S2. ^{1}H-NMR spectrum of PTB7-Th polymer.

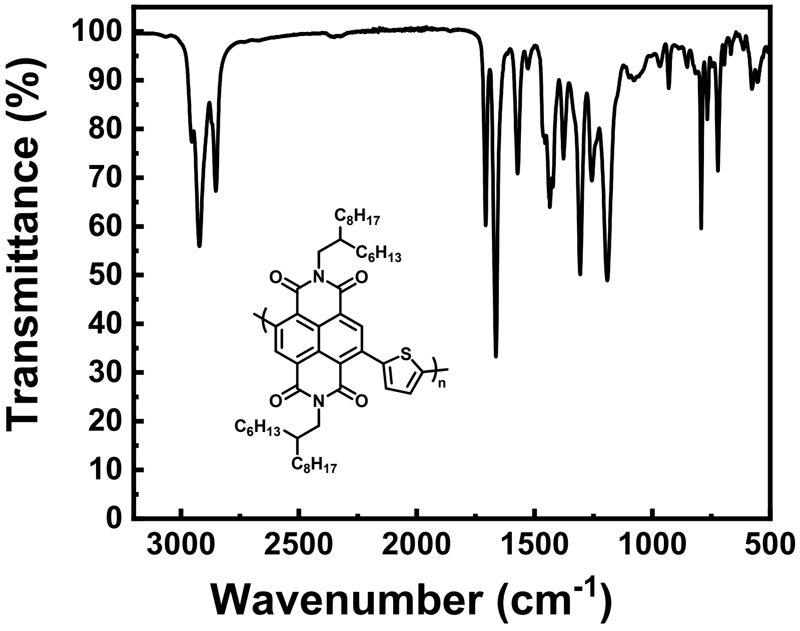

Figure S3. IR spectrum of PNDI2HD-T polymer.

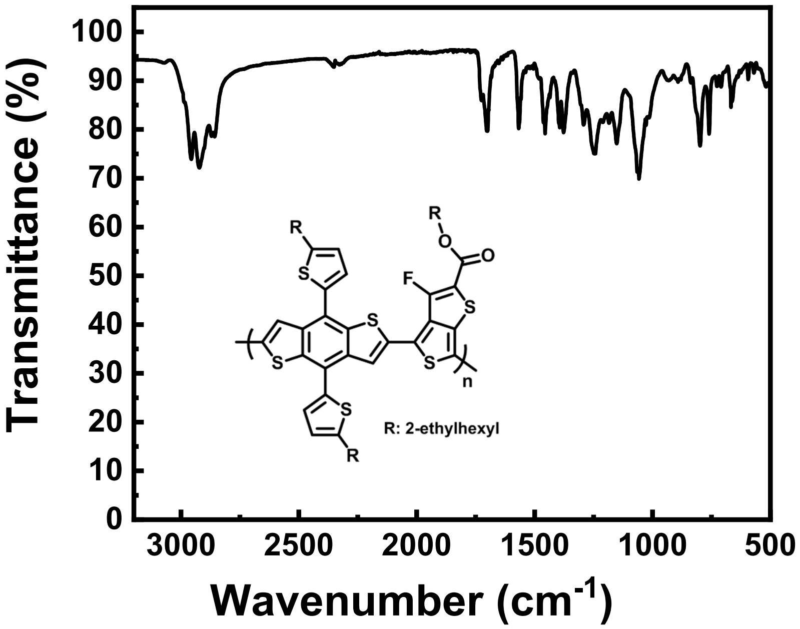

Figure S4. IR spectrum of PTB7-Th polymer.

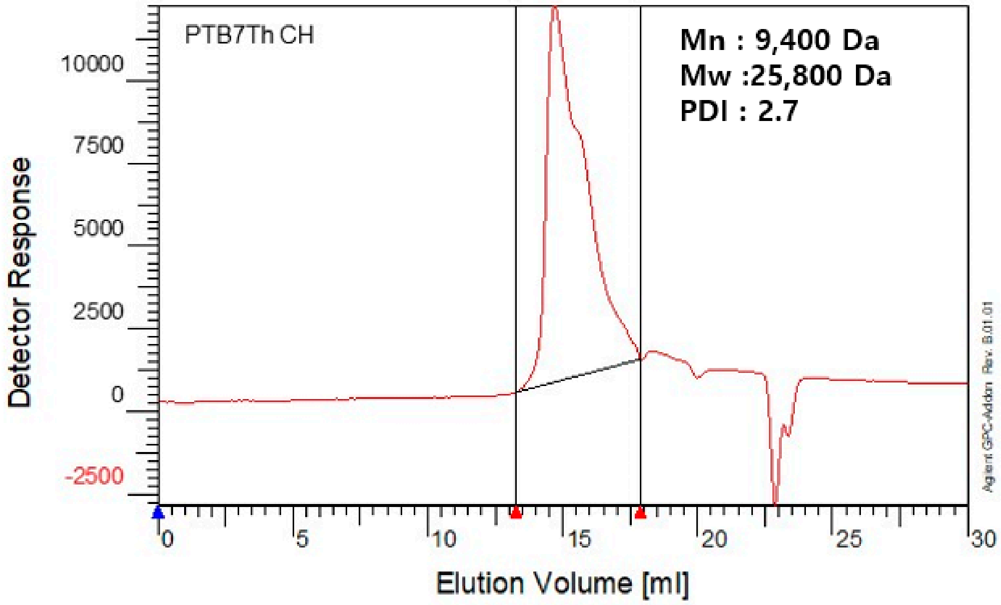

Figure S5. GPC data of cyclohexane batch of PTB7-Th.

Device fabrication and photovoltaic performance measurement

All-polymer solar cells were fabricated in an inverted structure of ITO/ZnO/PEIE/PTB7-Th:PNDI2HD-T/V_2O_5/Ag. ITO glass substrates were cleaned sequentially with isopropyl alcohol, acetone, and isopropyl alcohol for each 10 min in the ultrasonicator and dried in an oven. ITO glasses were then treated with ultraviolet/ozone for 20 min. ZnO precursor was prepared by dissolving 1 g of zinc acetate in 10 mL of 2-methoxyethanol containing 0.28 g of ethanolamine as a stabilizer. Then, the ZnO precursor solution was spin-coated on the cleaned ITO glass at 2,000 rpm for 40 s in N_2-filled glovebox and annealed at 200℃ on a hot plate in air for 45 min. ITO glass/ZnO substrates were transferred into a N_2-filled glovebox and a 0.2 wt% polyethylenimine (PEIE) solution

was spin-coated at 4,500 rpm for 40 s onto the ZnO layer and dried at 100℃ for 10 min inside glovebox. Active layer solution (PTB7-Th:PNDI2HD-T blend 1:1 wt/wt) containing various additives was spin-coated onto the PEIE layer. Finally, V_2O_5 (5.5 nm) and Ag (100 nm) layers were deposited by thermal evaporation and each active area was 0.125 cm^2.

Current (*J*)–voltage (*V*) characteristics of photovoltaic devices were measured using Keithley 2400 source measuring unit under AM 1.5G solar irradiation (100 mW/cm^2) with a mask at ambient condition. The light intensity of solar simulator (McScience, Suwon-si, Republic of Korea) was calibrated by NREL-calibrated silicon reference cell. EQE spectra were obtained in the wavelength range of 340 nm to 850 nm using incident photon-to-current conversion equipment (McScience, Suwon-si, Republic of Korea). Each wavelength photon intensity was calibrated by NIST-calibrated Si-photodiode reference cell. For a single kind of photovoltaic cell, more than 8 devices were fabricated and the best and averaged data from these devices were presented in the main text.

Transient photovoltage (TPV) measurements

TPV measurements were done under continuous illumination from white LED and perturbed the device using a pulsed green (520 nm) LED. TPV signals were acquired by a TDS3054B Tektronix digital oscilloscope. Data were fitted to an exponential decay function in order to find carrier lifetime.

$$\Delta V = V_{oc} + \Delta V_0 e^{-t/\tau} \quad (1)$$

where ΔV is the amount of the measured photovoltage, V_{oc} is the open-circuit voltage, ΔV_0 is the maximum incremented photovoltage under the pulsed green LED, and τ is the carrier lifetime.

Charge extraction (CE) measurements

CE measurements were done under perturbed white LED and its signals were acquired by a TDS3054 Tektronix digital oscilloscope. Current change was measured by monitoring voltage change through a 50 Ω resistor. Thus, voltage signals were integrated to find carrier density using following equation:

$$n = \frac{\#\, of\ carrier}{volume} = \frac{\int Idt}{q \times volume} = \frac{\int Vdt}{R \times q \times A \times L} \quad (2)$$

where n is the carrier density, R is 50 Ω, q is the charge of the electron, A is the photoactive layer area, and L is the photoactive layer thickness.

Space charge limited current (SCLC) measurements

Carrier mobilities of all-polymer blend films were measured by the space-charge-limited current (SCLC) method. Hole-only device structure was ITO/PEDOT:PSS/all-polymer blend film/MoO_3 (10 nm)/Al (100 nm). PEDOT:PSS was diluted with methanol as 1:1 volume ratio, which was spin-coated at 4,000 rpm for 40 s and thermally annealed at 110℃ for 15 min in air. MoO_3 and Al were thermally evaporated at 0.1 Å/s and 5 Å/s, respectively. Electron-only device structure was ITO/PEIE/ all-polymer blend film/Cs_2CO_3 (1 nm)/Al (100 nm). Cs_2CO_3 and Al were thermally evaporated at 0.1 Å/s and 5 Å/s, respectively. Current-voltage (*J-V*) curves were recorded in the range of -0.1 – 10 V and then found data corresponding SCLC regime which were fitted according to following equation:

$$J = \frac{9}{8}\varepsilon\varepsilon_0\mu\frac{V^2}{L^3} \quad (3)$$

J is current density, ε is the dielectric constant of the material, ε_0 is the permittivity of free space, μ is mobility, V is voltage, and L is thickness of active layer.

Photoactive film characterizations

AFM images were obtained using AFM5100N (HITACHI) with noncontact mode. UV-visible absorption spectra were obtained from spectrophotometer UV-2450 (SHIMADZU, Japan). For transmission electron microscopy (TEM) measurement blend films were prepared on PEDOT:PSS –coated Si wafer. PEDOT:PSS solution was diluted with methanol as 1:1 volume ratio, which was spin-coated at 4,000 rpm for 40 s and thermally annealed at 110℃ for 15 min in air. All-polymer blend films on PEDOT:PSS/Si wafer were floated off in deionized water and transferred on a carbon-coated copper grid. The images were obtained on a JEM-2100F (JEOL Ltd., Tokyo, Japan) operating at 120 kV, and the images were acquired with a SC 1000 CCD camera (Gatan, Warrendale, PA). For 2-Dimensional grazing incidence X-ray diffraction (2D GIXD) measurements, 0.1115 nm X-ray beam was incident on the polymer films at an angle of 0.12° and diffraction patterns were monitored by a Rayonix 2D SX165 detector. For 2D GIXD measurements, blend films were formed on PEDOT:PSS/Si wafer substrates prepared in the same way for TEM samples.

Table S1. Summary of GIXD parameters in the polymer films.

Polymer films	Peak	q (Å^{-1})	FWHM	d (Å)	L_c (nm)
PTB7-Th (P) XY	(100)	0.276	0.071	22.8	8.0
	(001)	0.856	0.057	7.3	9.9
	(010)q_y	1.704	0.318	3.7	1.8
	(010)q_z	1.623	0.256	3.9	2.2
PNDI2HD-T (N) XY	(100)	0.290	0.046	21.7	12.3
	(001)	0.624	0.052	10.1	10.9
	(010)q_y	1.707	0.605	3.7	0.9
	(010)q_z	1.526	0.376	4.1	1.5
Blend (B) XY	P(100)	0.276	0.046	22.8	12.3
	N(100)	0.290	0.090	21.7	6.3
	N(001)	0.629	0.045	10.0	12.6
	P(001)	0.853	0.047	7.4	12.1
	(010)q_y	1.691	0.339	3.7	1.7
	(010)q_z	1.574	0.368	4.0	1.5
Blend (B) XY+TMB	P(100)	0.276	0.051	22.8	11.0
	N(100)	0.290	0.087	21.7	6.5
	N(001)	0.625	0.042	10.1	13.6
	P(001)	0.855	0.066	7.3	8.5
	(010)q_y	1.771	0.327	3.5	1.7
	(010)q_z	1.566	0.315	4.0	1.8
Blend (B) XY+IN	P(100)	0.276	0.050	22.8	11.2
	N(100)	0.290	0.079	21.7	7.2
	N(001)	0.625	0.041	10.1	13.9
	P(001)	0.856	0.062	7.3	9.1
	(010)q_y	1.763	0.324	3.6	1.7
	(010)q_z	1.581	0.292	4.0	1.9
Blend (B) XY+TN	P(100)	0.276	0.043	22.8	13.0
	N(100)	0.290	0.072	21.7	7.9
	N(001)	0.620	0.039	10.1	14.5
	P(001)	0.852	0.062	7.4	9.2
	(010)q_y	1.756	0.347	3.6	1.6
	(010)q_z	1.581	0.289	4.0	2.0
Blend (B) XY+DPE	P(100)	0.276	0.038	22.8	14.8
	N(100)	0.290	0.066	21.7	8.5
	N(001)	0.617	0.028	10.2	20.0
	P(001)	0.855	0.058	7.4	9.8
	(010)q_y	1.756	0.311	3.6	1.8
	(010)q_z	1.596	0.272	3.9	2.1

Blend (B) XY+DBE	P(100)	0.276	0.047	22.8	12.0
	N(100)	0.290	0.074	21.7	7.6
	N(001)	0.626	0.042	10.0	13.5
	P(001)	0.856	0.062	7.3	9.1
	(010)q_y	1.745	0.386	3.6	1.5
	(010)q_z	1.587	0.303	4.0	1.9

Table S2. PL quenching efficiencies of XY and XY+ additive based polymer films.

**Excited at 700 nm**	**Area**		**PL quenching Efficiency (%)**
	***P* (PTB7-Th)**	***B* (Blend)**	
XY	6.725×10^{7}	3.498×10^{6}	94.8
XY+TMB	6.749×10^{7}	3.981×10^{6}	94.1
XY+IN	7.610×10^{7}	3.773×10^{6}	95.0
XY+TN	8.550×10^{7}	3.407×10^{6}	96.0
XY+DPE	9.196×10^{7}	4.194×10^{6}	95.4
XY+DBE	8.511×10^{7}	3.201×10^{6}	96.2
